# Clinical Features of Autoimmune Autonomic Ganglionopathy and the Detection of Subunit-Specific Autoantibodies to the Ganglionic Acetylcholine Receptor in Japanese Patients

**DOI:** 10.1371/journal.pone.0118312

**Published:** 2015-03-19

**Authors:** Shunya Nakane, Osamu Higuchi, Michiaki Koga, Takashi Kanda, Kenya Murata, Takashi Suzuki, Hiroko Kurono, Masanari Kunimoto, Ken-ichi Kaida, Akihiro Mukaino, Waka Sakai, Yasuhiro Maeda, Hidenori Matsuo

**Affiliations:** 1 Department of Clinical Research, Nagasaki Kawatana Medical Center, Nagasaki, Japan; 2 Department of Neurology, Nagasaki Kawatana Medical Center, Nagasaki, Japan; 3 Department of Neurology and Clinical Neuroscience, Yamaguchi University Graduate School of Medicine, Yamaguchi, Japan; 4 Department of Neurology, Wakayama Medical University, Wakayama, Japan; 5 Department of Neurology, Joetsu General Hospital, Niigata, Japan; 6 Department of Neurology, Saiseikai Kanagawa Prefecture Hospital, Kanagawa, Japan; 7 Division of Neurology, Department of Internal Medicine 3, National Defense Medical College, Saitama, Japan; 8 Department of Clinical Neuroscience and Neurology, Graduate School of Biomedical Sciences, Nagasaki University, Nagasaki, Japan; Istanbul University, TURKEY

## Abstract

Autoimmune autonomic ganglionopathy (AAG) is a rare acquired channelopathy that is characterized by pandysautonomia, in which autoantibodies to ganglionic nicotinic acetylcholine receptors (gAChR) may play a central role. Radioimmunoprecipitation (RIP) assays have been used for the sensitive detection of autoantibodies to gAChR in the serum of patients with AAG. Here, we developed luciferase immunoprecipitation systems (LIPS) to diagnose AAG based on IgGs to both the α3 and β4 gAChR subunits in patient serum. We reviewed the serological and clinical data of 50 Japanese patients who were diagnosed with AAG. With the LIPS testing, we detected anti-α3 and -β4 gAChR antibodies in 48% (24/50) of the patients. A gradual mode of onset was more common in the seropositive group than in the seronegative group. Patients with AAG frequently have orthostatic hypotension and upper and lower gastrointestinal tract symptoms, with or without anti-gAChR. The occurrence of autonomic symptoms was not significantly different between the seropositive and seronegative group, with the exception of achalasia in three patients from the seropositive group. In addition, we found a significant overrepresentation of autoimmune diseases in the seropositive group and endocrinological abnormalities as an occasional complication of AAG. Our results demonstrated that the LIPS assay was a useful novel tool for detecting autoantibodies against gAChR in patients with AAG.

## Introduction

The ganglionic nicotinic acetylcholine receptor (gAChR) mediates fast synaptic transmission in all peripheral autonomic ganglia (sympathetic, parasympathetic, and enteric ganglia) in the peripheral autonomic nervous system. AChRs on autonomic neurons are typically composed of two α3 subunits in combination with three other AChR subunits [1]. Although neurons of the autonomic ganglia can express numerous neuronal AChR subunits, including α3, α4, α5, α7, β2, and β4, the properties of the AChR at mammalian ganglionic synapses are most similar to AChRs that are formed by α3 and β4 subunits [2].

Autoimmune autonomic ganglionopathy (AAG) is an acquired immune-mediated disorder that leads to autonomic failure. The disorder is associated, at least in part, with autoantibodies to the gAChR. Antibodies to the gAChR that are found in the serum of 50% of patients with the acute or subacute form of AAG correlate with disease severity, and have been shown to be pathogenic [3,4]. Several studies have reported that these autoantibodies induce the internalization of cell-surface nicotinic gAChRs and thereby impair synaptic transmission [4,5]. Furthermore, it has been demonstrated that antibodies to the α3 subunit of the gAChR or AAG serum have been shown to directly cause autonomic dysfunction in experimental animal models of AAG [6,7]. Although these antibodies are proving to be useful serological markers of AAG, the positivity of gAChR antibodies in acute or subacute panautonomic failure remains around 50%. Cases of idiopathic pure autonomic neuropathy have been reported since 1975 in Japan, and 29 cases of AAG have been reported [8]. However, no assays are available that detect the antibodies to gAChR in Japan, and this has caused difficulty in the diagnosis of AAG. Furthermore, antibodies to non-α3 subunits, including the β4 gAChR subunit, have not been identified in AAG to date.

In this study, we attempted to develop a novel technique to detect the subunit-specific antibodies of gAChR without the use of a radioisotope. Here we established luciferase immunoprecipitation systems (LIPS) using GL^8990^ that can detect antibodies that bind to the α3 or β4 gAChR subunits with high sensitivity. The radioimmunoprecipitation (RIP) assay using [^125^I] labeled epibatidine has been used as a convenient method to detect autoantibodies to the gAChR [9]. In the RIP, a subunit-specific antibody cannot be detected because of the epibatidine binding the pentamer form of the gAChR. In contrast, LIPS, which is a powerful diagnostic technique for the serological testing of antibodies that are associated with many different human pathogens, is suitable for detecting a subunit-specific antibody [10–13]. In order to provide higher performance on the LIPS, we selected a *Gaussia* luciferase (GL) mutant, called GL^8990^, in this study. GL is the smallest marine luciferase that has been discovered [14]. GL generates a greater signal intensity from cells in culture (1000-fold) compared with the *Renilla* luciferase (RL) [15]. GL^8990^ (meaning F89W and I90L) is a GL mutant that is generated by site-directed mutagenesis and that emits bioluminescence that is 10 times stronger and/or prolonged than intact GL [16]. Here we performed the LIPS that with the α3 or β4 gAChR subunit fused to a luciferase to detect the respective autoantibodies in human sera. In addition, we extensively reviewed the histories and ongoing clinical and laboratory evaluations of 50 Japanese patients who had been diagnosed with AAG and measured their antibodies to gAChR with the LIPS. This study demonstrated the clinical features of AAG in patients in Japan and provides a tool for precise disease diagnosis.

## Materials and Methods

### Patients and serum samples

The series of subjects in this study was comprised of the groups of patients with AAG, healthy controls (HC), and controls with other diseases (DC). Serum samples from 50 patients with AAG were obtained from general and teaching hospitals throughout Japan between January 2012 and February 2014 (mean age, 52.5 ± 19.0 years old, 26 males and 24 females, Table 1). The clinical diagnoses were made in each hospital and the patients' clinical data were provided at the same time. Serum samples from patients showing limb muscle weakness or severe sensory disturbance were excluded from the study. The control groups consisted of 73 HC (mean age, 38.3 ± 11.1 years old, 31 males and 42 females) and 34 subjects with other diseases (DC: for details see S1 Table. Detailed clinical characteristics of OND patients; mean age, 56.3 ± 20.4 years old, 19 males and 15 females).

**Table 1:** Clinical features of patients with AAG/APD.

	Patients with AAG/APD	Patients with AAG/APD, Anti-gAChR Ab positive	Patients with AAG/APD, Anti-gAChR Ab negative	P value
Number of patients	50	24	26	
Age (yr)	52.5 ± 19.0	51.9 ± 20.4	53.0 ± 18.1	0.838
Age at onset (yr)	48.8 ± 20.1	46.8 ± 20.8	50.7 ± 19.7	0.495
Sex (female, %)	24 (48.0)	13 (54.2)	11 (42.3)	0.413
Duration of the autonomic symptoms (yr)	3.7 ± 6.9	5.1 ± 8.8	2.3 ± 4.1	0.344
Onset (%)	Subacute: 25 (50.0), Gradual: 25 (50.0)	Subacute: 9 (37.5), Gradual: 15 (62.5)	Subacute: 16 (61.5), Gradual: 10 (38.5)	0.095
Antecedent event (%)	11 (22.0)	4 (16.7)	7 (26.9)	0.212
Orthostatic hypotension and/or orthostatic intolerance (%)	42 (84.0)	20 (83.3)	22 (84.6)	0.915
Sicca complex (%)	28 (56.0)	14 (58.3)	14 (53.8)	0.760
Coughing episodes (%)	8 (16.0)	4 (16.7)	4 (15.4)	0.915
Heat intolerance and/or anhidrosis (%)	34 (68.0)	15 (62.5)	19 (73.1)	0.435
Pupil abnormality (%)	20 (40.0)	11 (45.8)	9 (34.6)	0.281
Gastrointestinal tract symptoms (%)	46 (92.0)	22 (91.7)	24 (92.3)	0.951
Bladder dysfunction (%)	29 (58.0)	16 (66.7)	13 (50.0)	0.242
Sexual dysfunction ^a^ (%)	15 (57.7)	7 (63.6)	8 (53.3)	0.628
Other clinical features ^b^ (%)	15 (30.0)	8 (33.3)	7 (27.0)	0.633
Complication: endocrine disorder ^c^ (%)	5 (10.0)	3 (12.5)	2 (7.7)	0.588
Complication: autoimmune disease ^d^ (%)	11 (22.0)	9 (37.5)	2 (8.0)	0.012
Complication: tumor ^e^ (%)	5 (10.0)	4 (16.7)	1 (3.8)	0.140

a. We reviewed the 26 male patients only.

b. Numbness, mental symptom, dementia, character change, and back pain

c. Amenorrhea, eating disorder, SIADH (Syndrome of inappropriate secretion of antidiuretic hormone), and panhypopituitarism

d. Still disease, PBC (primary biliary cirrhosis), Hashimoto disease, PMR (polymyalgia rheumatica), SLE (systemic lupus erythematosus), SS (Sjögren's syndrome), Graves’ disease, RA (rheumatoid arthritis), fibromyalgia, and other autoantibodies positive

e. Ovarian tumor, pancreas cancer, mediastinal tumor, and paranasal cancer

### Ethics

All of subjects gave their written, informed consent to participate in the present study. The study was approved by the Ethics Committee of Nagasaki Kawatana Medical Center (Nagasaki, Japan).

### LIPS assay for the detection of autoantibodies to gAChR

**Fig 1:**
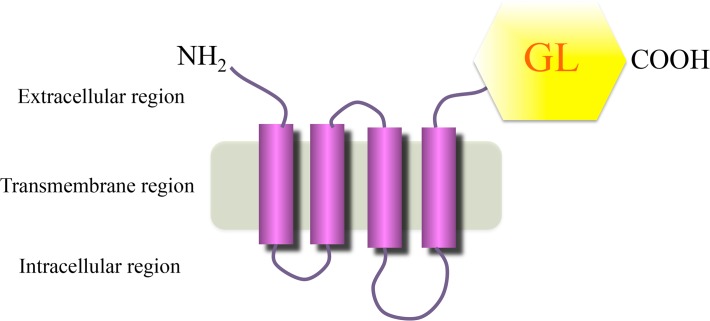
Schematic representation of the acetylcholine receptor (AChR) α3-*Gaussia* luciferase (GL) ^8990^. For the ganglionic AChR (gAChR)-LIPS assay, human embryonic kidney (HEK) 293 cells were transfected with an expression plasmid for the gAChRα3 or β4-GL reporter.

**Fig 2:**
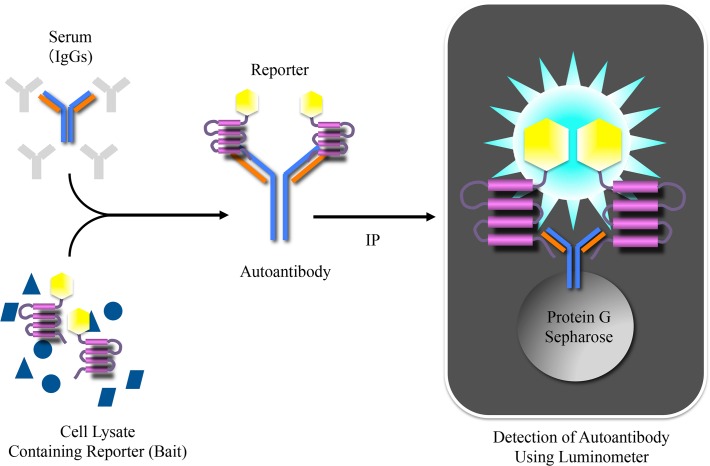
The Luciferase Immunoprecipitation Systems (LIPS). The soluble fractionated component from the solubilized HEK 293F cells, including the gAChRα3 or β4-GL, reacted with human serum, and the specific luciferase activities of the gAChRα3 or β4-GL were found with the luminometer. The *in vitro* LIPS assay can quantitatively evaluate an interaction between an antigen and an antibody with high sensitivity and without a radioisotope.

To generate luciferase reporters for the gAChR α3 and β4 subunits (termed gAChRα3-GL and gAChRβ4-GL, respectively) of the human gAChR, full-length human AChR α3 (P32297, Promega Corporation, Madison, WI, USA) or β4 (P30296, Promega Corporation) was fused to a *Gaussia* luciferase (GL) mutant (GL^8990^) (Fig. 1). Human embryonic kidney (HEK) 293F cells (Life Technologies Corportion, Graind Island, NY, USA) were transfected with the expression plasmid encoding the gAChRα3-GL or the gAChRβ4-GL with FuGENE6 (Promega Corporation). Two days later, the transfected cells were solubilized with a Tris-based saline containing 1% Triton^TM^ X-100. To detect the α3 or β4 gAChR antibodies, 100 μL of the soluble fraction, containing gAChR α3-GL or gAChR β4-GL, was incubated with 15 μL of human serum for 1 hour at 4°C. Subsequently, the fraction was mixed with 15 μL of protein G-sepharose (GE Healthcare, Little Chalfont, Buckinghamshire, UK) and 600 μL phosphate-buffered saline (PBS) with 3% bovine serum albumin and 0.05% Tween 20 and incubated for several hours at 4°C. Following centrifugation and washes with PBS containing 0.05% Tween 20 twice, the bioluminescence activities of the luciferase reporters in the protein G-sepharose were measured with a BioLux GL assay kit (New England Biolabs, Ipswich, MA, USA) and a Lumat LB 9507 luminometer (BERTHOLD TECHNOLOGIES GmbH & Co. KG, Bad Wildbad, Germany) (Fig. 2). The luminometer output was measured in relative luminescence units (RLU). In order to confirm the accuracy of the LIPS assay for the gAChR antibodies, we used commercially available antibodies to human gAChR α3 and β4 (H-100 and S-15; Santa Cruz Biotechnology, Inc., Dallas, TX, USA) as positive controls.

Based on the data for anti-gAChRα3 and β4 antibodies from the 73 HC, the cut-off values were calculated as the mean plus 3 standard deviations from the mean (SD). In this study, the antibody levels were expressed as an antibody index (A.I.) that was calculated as follows:

A.I. = [measurement value of the sample serum (RLU)]/[the cut-off value (RLU)]. The normal value that was established in this study from healthy individuals was <1.0 A.I.

### The RIP assay for the detection of autoantibodies to gAChR

Sera from eight of the 50 patients with AAG had been examined previously for antibodies to the gAChR with conventional RIP in the laboratory of Dr. Vernino. These assays for antibodies against the AChR were performed as previously described. In brief, the antibodies were detected with an immunoprecipitation assay in which the AChR antigen was solubilized from a human neuroblastoma cell line (IMR-32) and complexed with iodine I^125^-labeled epibatidine [3,4,17].

Our assay for the anti-gAChR antibodies may have a different sensitivity and specificity compared with the RIP assay; therefore, we were able to compare the results of the two different assays on these eight samples.

### Clinical assessment of autonomic function

Patients with generalized or restricted autonomic dysfunction were identified in each participating hospital in Japan. All of the patients with AAG had dysfunction in at least one autonomic domain, and they underwent a baseline assessment, which included a determination of the gAChR α3 and β4 antibody levels. Subacute onset was defined as the reaching of the peak of autonomic failure within 3 months, and chronic was defined as reaching of the peak after 3 months. Comprehensive clinical, hematologic, biochemical, neurological, and serologic assessments of all patients were performed at baseline. In addition, cerebrospinal fluid analysis was also conducted.

We inquired about the presence or absence of each of the following functions that are controlled by the autonomic system: syncope or orthostatic hypotension for orthostatic intolerance; sicca complex, dryness of the skin, or hypohidrosis/anhidrosis for heat intolerance; pupillary dysfunction; diarrhea or constipation for dysfunction of the gastrointestinal system; dysuria or urinary retention needing catheterization for bladder dysfunction; and sexual dysfunction. However, we were not able to assess the extent of each autonomic symptom rigorously with the composite autonomic scoring scale. Patients with known causes of autonomic failure, including multiple system atrophy, diabetes, and amyloidosis were excluded.

Each patient went through autonomic testing, which involved the Schellong test, head-up tilt test, measurement of the coefficient of variation in R-R intervals (CV_R-R_), noradrenaline (NA) infusion test, pupillary response to local instillation, assessment of the plasma levels of catecholamines, sweat testing, quantitative sudomotor axon reflex test (QSART), [^123^I] meta-iodobenzylguanidine (^123^I-MIBG) myocardial scintigraphy, and cystometry. The physiologic analog of noradrenaline, ^123^I-MIBG, traces the uptake and transport in both noradrenaline presynaptic sympathetic nerve terminals and in subsequent vesicular storage [18]. Postganglionic presynaptic cardiac sympathetic nerve endings can be noninvasively assessed by MIBG scintigraphy because a reduction in cardiac MIBG uptake (H/M ratio) indicates postganglionic sympathetic dysfunction. Cardiac MIBG uptake is reduced in patients with Lewy body diseases such as Parkinson’s disease, as well as dementia with Lewy bodies [19,20]. In the standard procedure, the H/M ratio is calculated on early and delayed anterior chest planar images by drawing a region of interest including the heart (H) and the other one over the upper mediastinum (M). However, we were unable to unify the itemss of autonomic testing facilities among the different hospitals.

### Statistical analysis

Commercially available statistics software was used for the data analysis (SigmaPlot, Systat Software, Inc., San Jose, CA, USA). The A.I. data that were normally distributed were analyzed with one-way analysis of variance. For the data that were not normally distributed, a one-way analysis of variance of ranks was employed. The significance level was set at P<0.05.

## Results

### Establishment of the LIPS assay for the detection of antibodies to the gAChR

**Fig 3:**
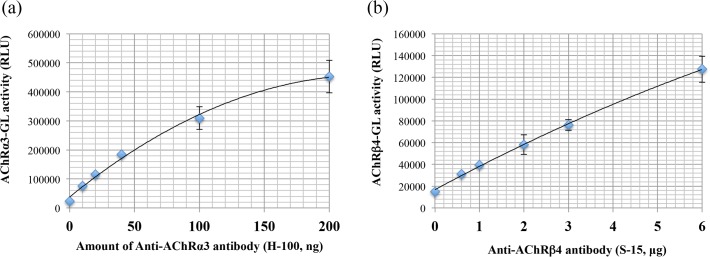
Confirmation of the LIPS assay system for the gAChRα3 or β4 with ready-made antibodies. The anti-gAChRα3 antibody (H-100) and the anti gAChRβ4 antibody (S-15) bound the gAChRα3-GL and the gAChRβ4 reporters, respectively in a dose-dependent manner (a and b). The X-axis indicates the amount of ready-made gAChRα3 or β4 antibody used. The Y-axis indicates the gAChRα3 or β4-GL activity. The line with closed diamonds shows the results that were obtained in this experiment.

The RIP is a very useful tool for obtaining information about the total amount of antibodies to gAChR, but it cannot distinguish subunit-specific antibodies [3]. In order to detect subunit-specific antibodies for the gAChR, we prepared two subunit-specific luciferase reporters, termed gAChRα3-GL and gAChRβ4-GL. In order to confirm that these subunit-specific luciferase reporters work as a bait in the LIPS, we examined a LIPS assay that used ready-made gAChR subunit-specific antibodies. As shown In Fig. 3, there is a dose-dependent response was observed between the amount of ready-made gAChR subunit-specific antibody and the gAChR subunit-specific luciferase reporter activity. These data demonstrate that each gAChR subunit-specific antibody properly bounds gAChRα3-GL or gAChRβ4-GL.

### Detection of the autoantibodies to the gAChR in patients with AAG

**Fig 4:**
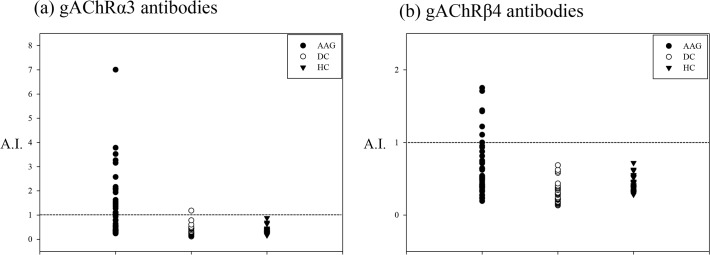
LIPS for gAChR in the sera from patients with autoimmune autonomic ganglionopathy (AAG) and controls. We tested the sera from patients with AAG, disease controls (DC), and healthy controls (HC). a) Anti-gAChRα3 antibodies were detected in 23 samples. The mean anti-gAChRα3 antibody level in the HC was 0.305 antibody index (A.I.), which was significantly lower than in the AAG samples with a mean level of 1.210 A.I. (*p* < 0.001). b) Anti-gAChRβ4 antibodies were also detected in seven samples, as shown in Fig. 4B (*p* = 0.005). The mean anti-gAChRβ4 antibody level in the HC was 0.367 A.I., which was significantly lower than the measn level of 0.618 A.I. in the AAG samples (*p* < 0.001).

Both the anti-gAChRα3 and anti-gAChRβ4 antibodies were determined by the LIPS assay to be present in 0.0% (0 of 73) of the HC. In contrast, 48% (24 of 50) of the sera from patients with AAG were positive for autoantibodies (*p* < 0.001, Fig. 4A). The anti-gAChRα3 antibodies were detected in 23 samples and the anti-gAChRβ4 antibodies were detected in seven samples (14.0%), as shown in Fig. 4B (*p* < 0.001). Both of the antibodies were detected in six samples. The mean anti-gAChRα3 antibody levels in the HC and the DC were 0.305 A.I. and 0.336 A.I., respectively. These levels were significantly lower than the mean level in the AAG samples with a mean level of 1.210 A.I. (*p* < 0.001, Fig. 4A). Similarly, the mean anti-gAChRβ4 antibody level in HC was 0.367 A.I. and DC was 0.302 A.I., respectively. Those were significantly lower than the AAG samples with a mean level of 0.618 A.I. (*p* < 0.001, Fig. 4B). In the DC group, we detected anti-gAChRα3 antibodies in the serum of the patient with the suspected case of amyloid neuropathy.

### Clinical profile of the anti-gAChR antibody-positive and -negative patients with AAG

The clinical characteristics of the patients are presented in Table 1. The age at the onset and the duration of the autonomic symptoms were 48.8 ± 20.1 (mean ± standard deviation) years and 3.7 ± 6.9 years, respectively. The patterns of mode were divided into subacute and gradual groups, according to the duration to the peak of autonomic symptoms. Half of the patients with AAG had subacute onsets and half had chronic progressive presentations. Gastrointestinal tract symptoms were the most frequently observed (92.0%). Table 1 compares the clinical features between patients who were positive and those who were negative for the anti-gAChR antibodies. Gradual onset was more common in the anti-gAChR antibody-positive patients than in the antibody-negative patients (62.5% *vs*. 38.5%). No significant differences in the clinical findings were noted, except for a higher frequency of other autoimmune disease complications in the patients who were positive for the anti-gAChR antibody compared to the antibody-negative patients (37.5% *vs*. 8.0%, *p* = 0.012).

### Clinical characteristics and autonomic symptoms of the patients with AAG who were anti-gAChR antibody-positive (Table 2)

Table 2 summarizes the clinical characteristics and autonomic symptoms of the patients with AAG. An antecedent event was reported in four patients shortly before the initiation of autonomic symptoms. In our study, major autonomic symptoms, including orthostatic, sicca, sudomotor, papillary, gastrointestinal, and urinary symptoms were analyzed. Orthostatic hypotension for orthostatic intolerance and gastrointestinal tract symptoms were observed in 20 (83.3%) patients and 22 (91.7%) patients, respectively. Gastrointestinal tract symptoms were composed of various digestive system problems, such as constipation (n = 14), early satiety (n = 4), vomiting (n = 4), abdominal pain (n = 4), anorexia (n = 4), diarrhea (n = 4), ileus (n = 3), alternate stool abnormality (n = 3), taste impairment (n = 1), and achalasia (n = 3). Pupillary dysfunction was observed in 11 patients, including two patients who had Adie’s tonic pupil. The initial symptoms of seropositive AAG/acute pandysautonomia (APD) in 15 patients (62.5%) were orthostatic hypotension, involving lightheadedness, orthostatic intolerance, or syncope. The autonomic manifestations of the anti-gAChR antibody-positive patients were widespread, and they affected both sympathetic and parasympathetic functions. However, 3 patients (Patient 6, 16, and 17) had only one symptom (disturbance of the digestive system, bladder dysfunction, and orthostatic hypotension, respectively). Patient 6 had a history of recurrent ileus and severe abdominal pain. She previously had an operation to remove a sigmoid volvulus 1 year before the onset of repeated ileus and abdominal pain. As for the other symptoms, attacks of coughing were observed in four patients (16.7%), and six patients (Patients 1, 5, 11, 12, 14, and 15) had a subjective numbness or superficial sensory disturbance in the extremities or trunk. Psychiatric symptoms were observed in three patients (Patient 8, 9, and 14). Patient 8 demonstrated infantilization after the onset of autonomic symptoms and had frequent syncope with emotional strain. Patient 9 showed memory disturbances and apathy in daily living, such as domestic duties. Patient 14 also demonstrated character changes, such as the tendency to act in a childish manner. Three patients with the following endocrine disorders: amenorrhea (Patient 4), syndrome of inappropriate secretion of antidiuretic hormone (SIADH) (Patient 14), and panhypopituitarism (Patient 15). Nine patients presented the other following autoimmune diseases: rheumatoid arthritis (n = 2), primary biliary cirrhosis (n = 2), secondary Sjögren's syndrome (n = 2), Still disease (n = 1), Hashimoto disease (n = 1), polymyalgia rheumatica (n = 1), systemic lupus erythematosus (n = 1), Graves’ disease (n = 1), and fibromyalgia (n = 1).

**Table 2:** Clinical and autonomic characteristics at baseline of anti-gAChR Ab positive AAG patients.

Patient	Age	Sex	Onset age	Duration	Onset ^a^	AE ^b^	OH, OI	Sicca	Coughing episodes	HI, AH	Pupil abnormality ^c^	GI ^d^	Bladder dysfunction	Sexual dysfunction	Other clinical features	Complication: endocrine disorder, autoimmune disease	Complication: tumor
1	75	M	59	16	Gradual	−	**+**	+	−	+	+	+	+	+	Numbness	−	−
2^[^ ^53^ ^]^	60	M	60	0	Subacute	+	**+**	+	−	+	+	+	+	−	−	Still disease susp.	−
3	39	F	39	0	Subacute	−	**+**	+	−	−	−	+	−		−	ANA positive	−
4	26	F	21	5	Gradual	−	−	**+**	−	+	+	+	+		−	Amenorrhea	Ovarian tumor
5	68	M	53	15	Gradual	−	**+**	−	−	−	−	+	+	+	Numbness	−	−
6	37	F	35	2	Gradual	−	−	−	−	−	−	**+**	−	−	−	−	−
7	45	M	45	0	Subacute	+	+	+	−	+	+	+	+	+	−	−	−
8	60	M	60	0	Subacute	−	**+**	+	−	+	+	+	+	+	Mental symptom	−	−
9	79	F	77	2	Gradual	−	**+**	−	−	−	+	+	+		Dementia	PBC, Hashimoto dis.	−
10	78	M	78	0	Subacute	−	**+**	−	−	+	−	+	−	−	−	−	−
11	67	M	59	8	Gradual	−	**+**	+	−	−	−	+	−	−	Numbness	−	−
12	49	M	12	37	Gradual	−	**+**	+	+	+	+	+	+	+	Sensory disturbance	−	−
13	73	F	66	7	Gradual	−	**+**	+	−	+	−	+	+		−	−	Mediastinal tumor
14	16	M	16	0	Subacute	−	+	−	+	**+**	+	+	−	−	Numbness, character change	SIADH	−
15	56	M	55	1	Gradual	−	**+**	−	+	+	−	+		+	Numbness	PMR, panhypopituitarism	−
16	54	F	35	19	Gradual	−	−	−	−	−	−	−	**+**		−	−	−
17	84	F	84	0	Gradual	−	+	−	+	−	−	+	+		−	−	Paranasal cancer
18	37	F	37	0	Subacute	−	**+**	+	−	+	+	+	+		−	SLE, SS	−
19	38	F	39	0	Subacute	−	**+**	−	−	−	−	−	−		−	Graves' dis.	Ovarian tumor
20	68	F	66	2	Gradual	−	**+**	+	−	+	−	+	+		−	RA, SS	−
21	46	F	39	7	Gradual	+	**+**	−	−	+	−	+	+		−	RA, fibromyalgia	−
22	49	F	47	2	Gradual	−	−	+	−	−	−	**+**	−		−	PBC	−
23	6	F	6	0	Subacute	+	+	+	−	+	+	**+**	+		−	−	−
24	36	M	35	0.5	Gradual	−	+	+	−	+	+	**+**	+	+	−	−	−
																	

Initial symptoms were expressed in bold.

AE = antecedent event; OH = orthostatic hypotension; OI = orthostatic intolerance; HI = heat intolerance; AH = anhidrosis; GI = gastrointestinal tract symptoms; α3 Ab = ganglionic acetylcholine receptor α3 antibody; β4 Ab = ganglionic acetylcholine receptor β4 antibody; A.I. = Antibody Index; AE = antecedent event; OI = orthostatic intolerance; OH = orthostatic hypotension; HI = heat intolerance; AH = anhidrosis; GI = gastrointestinal tract symptoms; Dept = department

a. Subacute = peak of autonomic failure within 3 months; gradual = gradual onset of chronic autoimmune autonomic ganglionopathy with the peak of autonomic failure after 3 months.

b. Patient 2 = fever up; 7 = epididymitis; 21 = influenza virus type A infection; 23 = fever up and cough.

c. Patient 4 and 7 = Adie’s tonic pupil. The other cases had the abnormality of papillary reflex to light bilaterally or unilaterally.

d. Patient 1 = constipation; 2 = constipation; 3 = early satiety and vomiting; 4 = constipation; 5 = constipation; 6 = constipation, ileus, and sigmoid volvulus suspected; 7 = early satiety, vomiting, alternate stool abnormality, abdominal pain, and taste impairment; 8 = constipation; 9 = constipation; 10 = constipation; 11 = anorexia and diarrhea; 12 = diarrhea and achalasia; 13 = constipation, early satiety, vomiting, and ileus; 14 = abdominal pain, anorexia, and diarrhea; 15 = diarrhea; 17 = constipation, anorexia, and achalasia; 18 = alternate stool abnormality, abdominal pain, and anorexia; 20 = constipation; 21 = constipation; 22 = early satiety, ileus, alternate stool abnormality, and abdominal pain; 23 = constipation, vomiting, and achalasia; 24 = constipation.

### Autonomic function studies of the anti-gAChR antibody-positive patients with AAG

Table 3 shows the results of the autonomic function studies of the anti-gAChR antibody-positive patients. Some information was missing because some of the hospitals did not have adequate equipment for the autonomic function tests. Of these 24 patients, abnormalities the orthostatic changes in systolic blood pressure and the CV_R-R_ were observed at high rates (86.4% and 75.0%, respectively). Cardiac MIBG uptake (H/M ratio), which was measured by ^123^I-MIBG myocardial scintigraphy in 11 anti-gAChR antibody-positive patients, was decreased in nine patients (81.8%). Urodynamic studies were performed in five of 24 patients. Residual urine of more than 50 mL was noted in four patients (80.0%). Sudomotor and cutaneous vasomotor functions were assessed by thermoregulatory sweat testing in three patients and a reduced ability to sweat was reported in two patients. Plasma norepinephrine values were measured in seven of 24 patients. Six had reduced supine norepinephrine below 100 pg·mL^-1^ (85.7%). Pupillary responses to 1% phenylephrine, 5% tyramine, and 5% cocaine were examined in two patients (Patients 2 and 14) and 0.1 to 0.125% pilocarpine in two patients (Patients 4 and 7) in a totally dark room. In the former test, remarkable mydriasis was observed as a pupillary response to the local instillation of 1% phenylephrine. In the latter test, the affected Adie’s pupil reacted with miosis to low dose pilocarpine. We confirmed albuminocytologic dissociation in seven of 12 patients (58.3%) in a cerebrospinal fluid analysis.

**Table 3:** Autonomic function tests at baseline of anti-gAChR Ab positive AAG patients.

Patient	Age	Sex	Anti-gAChRα3 Ab (A.I., LIPS)	Anti-gAChRβ4 Ab (A.I., LIPS)	Anti-gAChR Ab (nmol/L, RIP)	OH in HUTT	Decreased CV R-R	Decreased H/M ratio	Residual urine in urodynamic study	Abnormality of TST	Reduction of resting plasma NE	Abnormality of pupillary response	Albuminocytologic dissociation in CSF
1	75	M	1.626	1.000	0.060	+	+	+					−
2 ^42)^	60	M	2.163	0.389	1.220	+	+		+		+	+	
3	39	F	1.000	0.638			−				−		
4	26	F	3.520	0.376		+	+			+	+	+	
5	68	M	2.046	1.219		+	−	+	+				+
6	37	F	1.352	0.636									
7	45	M	7.005	1.443	42.160	+	+		+		+	+	−
8	60	M	1.008	0.494		+	+			+	+		
9	79	F	1.027	0.935		+	+	+					−
10	78	M	1.419	0.737		+	+	+					−
11	67	M	3.265	1.107		+	+	+					+
12	49	M	0.937	1.428		+							
13	73	F	1.103	0.877		+	+	+					+
14	16	M	1.488	0.713	negative	+	+	+	+		−	+	+
15	56	M	3.781	1.708		+	+						+
16	54	F	2.084	0.525									
17	84	F	2.573	1.752		+	+	+					
18	37	F	1.054	0.523		+	−	+			+		+
19	38	F	2.101	0.488		+	+				+		−
20	68	F	1.542	0.952		+	+						
21	46	F	1.113	0.478		−	−	−		−			
22	49	F	3.154	0.336	0.100	−		−	−				
23	6	F	1.275	0.456		+	−						+
24	36	M	1.936	0.323		+	+						

Anti-gAChRα3 Ab = ganglionic acetylcholine receptor α3 antibody; Anti-gAChRβ4 Ab = ganglionic acetylcholine receptor β4 antibody; A.I. = Antibody Index; OH = orthostatic hypotension; HUTT = head-up tilt test; CV R-R = CV = coefficient of variation R-R interval; H/M ratio = heart-to-mediastinum ratio in ^123^I-MIBG myocardial scintigraphy; TST = thermoregulatory sweat test; NE = norepinephrine; SSR = sympathetic skin response; QSART = quantitative sudomotor axon reflex test; CSF = cerebrospinal fluid

### Illustrative cases

**Fig 5:**
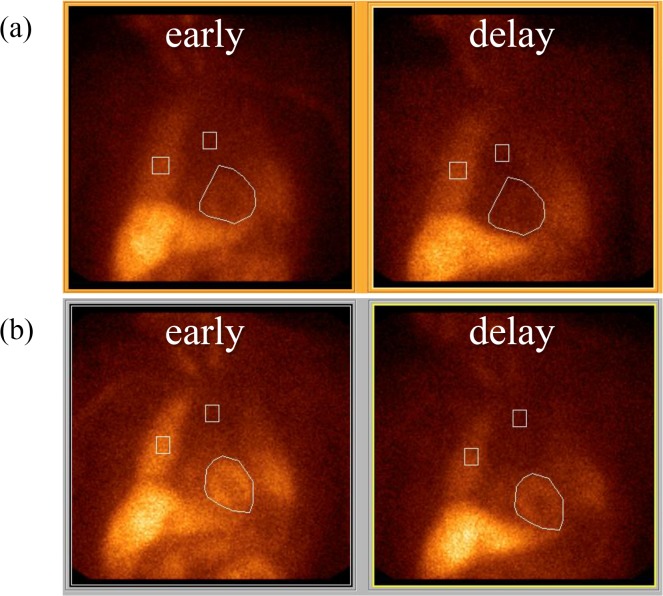
[^123^I] meta-iodobenzylguanidine (MIBG) cardiac imaging. Early and Delayed. a)^123^I-MIBG myocardial scintigraphy revealed that the heart/mediastinum (H/M) ratio was decreased at the baseline (early, 1.55; delay, 1.32). A reduced HM ratio indicates peripheral noradrenergic depletion. b) Combined immunomodulatory therapies resulted in a remarkable improvement in the H/M ratio (early, 2.38; delay, 2.23).

Patient 1: A 75-year-old man who had been affected by AAG for at least 15 years and who had severe orthostatic hypotension (supine, 102/64 mmHg; sitting, 60/34 mmHg). He had recurrent syncope (8–10 times a day), dry mouth, heat intolerance, severe constipation, and great difficulty in passing urine (he required intermittent self-catheterization). An examination confirmed anisocoria and numbness in the all of his extremities. The serum levels of the anti-gAChR autoantibodies were 1.626 A.I. (α3) and 1.000 A.I. (β4). ^123^I-MIBG myocardial scintigraphy revealed a decreased H/M ratio (early, 1.554; delay, 1.320, Fig. 5A). The normal values for the H/M range were from 1.9 to 2.8 with a mean of approximately 2.2. Based on the basis of the diagnosis of AAG, he was treated with two doses of intravenous methylprednisolone (IVMP), which was followed by intravenous immunoglobulin (IVIg), in order to reach a sustained improvement of the autonomic symptoms. He maintained the improvements in the orthostatic hypotension and experienced complete recovery of gastrointestinal function with IVMP and IVIg, which was followed by oral prednisolone. The levels of the anti-gAChR autoantibodies in Patient 1 converted to normal values after treatment (α3, 0.727 A.I.; β4, 0.473 A.I.), and treatment resulted in a remarkably improvement of H/M ratio (early, 2.380; delay, 2.231, Fig. 5B).

Patient 4: A 25-year-old woman who had been affected by AAG for at least 5 years and who had severe constipation. She developed orthostatic intolerance, dry mouth and eye (left), photophobia, and reduced sweating on the right side of the face. An examination revealed Adie’s tonic pupil (left), loss of deep tendon reflexes in the four extremities, preserved sensations, and normal muscle strength. We confirmed the orthostatic hypotension (supine, 108/58 mmHg; standing, 75/32 mmHg). Autonomic function tests confirmed generalized autonomic failure. She had developed several autonomic symptoms since the first episode of amenorrhea at the age of 20. The level of the gAChRα3 antibody was 3.520 A.I. She was treated with plasma exchange (PE) and showed improvements in orthostatic intolerance, deep tendon reflexes, and Adie’s tonic pupil in response to PE that was followed by oral prednisolone. Surprisingly, menstruation started again after a series of immunotherapy. The level s of the anti-gAChRα3 antibody remained positive in this patient, but with a substantially reduced (1.151 A.I.).

### Comparison of the RIP and the LIPS assay for the detection of the autoantibodies to the gAChR

Eight samples from patients who were previously measured for gAChR antibodies by the RIP were also evaluated by the LIPS assay. Seventy-five percent (6/8) of the samples demonstrated perfect agreement on both assays. Anti-gAChRα3 antibodies were detected in Patient 14 by the LIPS assay, but this sample was seronegative in the RIP (Table 3). Another sample demonstrated an opposite pattern. We further evaluated the correlation between the level of the anti-gAChRα3 antibody that was measured by LIPS and the level of the anti-gAChR antibody that was measured by the RIP in four seropositive samples. Some of the samples tracked each other well between the two assays.

## Discussion

Since the gAChR was identified as an autoantigen that is associated with the pathogenesis of AAG, it has been a challenge to search for gAChR-specific autoantibodies. RIA and cell-based assays (CBA) have been available for the detection of autoantibodies to the gAChR in the sera of patients with AAG [1,5]. In this study, we established the LIPS assay for the detection of gAChR antibodies and examined AAG cases with subunit-specific antibodies for gAChR in Japan for the first time.

For the the detection of subunit-specific antibodies to the ion-channels, Ching et al. have reported that the use of the LIPS method to detects the subunit-specific autoantibody of the AChR in patients with myasthenia gravis [21]. Myasthenia gravis (MG) is an autoimmune channelopathy that is caused by autoantibodies to the apparatus of the neuromuscular junctions (NMJs). In about 80% of the patient sera, autoantibodies to the muscle-type of the nicotinic AChR are detected [22]. It has been known that the AChR is a pentamer that is composed of four subunits (α1, β1, γ or δ, and ε). Ching et al have demonstrated that in LIPS using the α1 AChR subunit that is fused to *Renilla* luciferase in LIPS was partly useful for the detection of subunit-specific antibodies of the AChR. In the present study, gAChR autoantibodies were detected by LIPS in about 48% of the patients with AAG, which indicated that our data on the frequency of the gAChR autoantibodies matched the results of the previous study that used the RIP. Furthermore, the levels of the gAChR antibody that were estimated by LIPS (the current group) and the RIP (reported by studies from the Steven Vernino Laboratory and Mayo Medical Laboratories [Patient 1, 2, 7, 14, and 22, as shown in Table 3]) showed a similar tendency. Together, these results demonstrated that the LIPS exhibits a similar performance to the RIP, at least in the detection of autoantibodies to gAChR. Needless to say there is still a slight possibility that a pentamer-specific antibody exists in the AAG patient serum, because these results matched incompletely. In addition, we have identified a subunit-specific antibody for the gAChR, the anti-AChRβ4 antibody for the first time. Another subunit specific antibody for gAChR, anti-AChRα3 antibody, causes several autonomic dysfunctions in an experimental AAG model [6,7,23]. However, it is unclear whether the anti-AChRβ4 antibody is involved in the pathogenesis of AAG. Further studies are necessary to prove this, and we are planning to test the autoantibodies for other subunits of the nicotinic AChR (e.g., α5, β2).

AAG has two patterns of onset. In the present study, a gradual mode of onset was more common in the seropositive group than in the group that was seronegative for anti-gAChR antibodies, although Sandroni et al. have reported that subacute onset was more common in the seropositive group [24]. There was no difference between the demographic features of the seropositive patients in gradual onset and subacute AAG in seropositive patients (S2 Table. Demographic features of patients with subacute AAG/APD and gradual AAG/APD) and no relationship between antibody status and the temporal profile. Subacute AAG was often preceded by an illness that was presumed to be a viral or bacterial infection. Patients with chronic AAG with mild or restricted autonomic failure usually present with low antibody levels whereas high levels of antibodies are associated with severe acute/subacute AAG subtypes. However, the highest A.I., which was found in Patient 7, was associated with chronic AAG and severe autonomic dysfunction. These findings suggested that it is necessary to compile information about patients with AAG more precisely. The main findings of the clinical and autonomic characteristics were that patients with AAG were associated with a high rate of orthostatic hypotension for orthostatic intolerance and upper and lower gastrointestinal tract symptoms in both the seropositive and seronegative group. There was no significant difference in the occurrence of autonomic symptoms between the seropositive and seronegative group. It remains possible that unknown autoantibodies and other causative agents exist in the seronegative group. Clinically, it is difficult to distinguish the chronic form of seronegative AAG from other degenerative disorders of the autonomic nervous system (e.g., pure autonomic failure). Of note, three patients had achalasia in the seropositive group. The pathogenesis of achalasia remains unclear, but anti-gAChR antibodies might contribute to it. All of the anti-gAChR antibody-positive patients, except for Patients 6, 16, and 19, presented pandysautonomia. Patient 6 had been diagnosed with a chronic intestinal pseudo obstruction (CIPO) before being testing with the autoantibodies. Our data were consistent with prior reports that showed that patients with limited autonomic symptoms demonstrated a relatively lower A.I. Sandroni et al. have previously reviewed other autonomic neuropathies that are associated with the gAChR antibody. Occasional positivity to gAChR antibodies has been found in postural orthostatic tachycardia, CIPO, chronic idiopathic anhidrosis, and distal small fiber neuropathy [25]. All of these were listed as possible clinical phenotypes of anti-gAChR autoimmunity, although they show low antibody levels. The results of our study indicated a similar tendency and suggested a possible correlation between antibody levels and the phenotype of the dysautonomia. In the present study, however, we were not able to perform a correlational analysis between the level of A.I. levels and the clinical severity of the autonomic symptoms because we did not perform a quantitative assessment for autonomic dysfunction. Moreover, several research groups have reported that patients with other neuroimmunological disorders (myasthenia gravis, Lambert-Eaton myasthenic syndrome, Guillain-Barré syndrome, and chronic inflammatory demyelinating polyneuropathy) have autoantibodies to gAChR and autonomic symptoms [26–28]. We need to verify the pathogenicity of these autoantibodies and the correlation between antibody levels and disease severity.

The Patients with AAG in Japan were younger and more male predominant than those in Western countries [8]. In the present study, Japanese patients with AAG also had a lower age at onset. The seronegative and seropositive groups did not differ significantly in age and gender, although the seropositive group showed a female predominance. Of the 29 Japanese patients previously reported, 10 (34.5%) had coughing episodes and 12 (41.4%) had psychiatric symptoms. The present study found several anti-AChR antibody-positive patients with coughs (Patients 12, 14, 15, and 17) and psychiatric symptoms (Patients 8, 9, and 14), but these patients had a low frequency when compared with the findings of previous reports. Baker et al. reported encephalopathy co-occurring with classic subacute AAG [29]. They revealed the presence of antibodies that were directed against both the central α4 and α7 nAChRs in the serum of the patient. That study is the first case report of patients with AAG presenting with antibodies directed against both peripheral and central nAChRs. Watson et al. found antibodies that specifically blocked the function of α7 nAChRs in patients with Rasmussen encephalitis [30]. Therefore, it may be necessary to establish a LIPS assay for the detection of autoantibodies to these nAChRs. Six AAG anti-gAChR antibody-positive patients (Patients 1, 5, 11, 12, 14, and 15) complained of subjective numbness as an extra autonomic symptom, and the numbness might have been caused by extensive disturbance of the sympathetic and parasympathetic nervous system. However, this is an important symptom that may distinguish AAG from other disorders such as acute autonomic sensory neuropathy, Guillain-Barré syndrome, and chronic inflammatory demyelinating polyneuropathy. A nerve conduction study could be performed to assess this, and a nerve biopsy may be necessary in the patient with AAG.

The seropositive group had a significant overrepresentation of autoimmune diseases. Autonomic dysfunctions have been reported in association with Sjögren’s syndrome [31,32], systemic sclerosis [33], systemic lupus erythematosus [34], rheumatoid arthritis [35,36], and mixed connective-tissue disease [37]. Although AAG and the other autoimmune diseases can coexist due to the same background of autoimmunity, few reports have referred to anti-gAChR antibodies in these autoimmune diseases. In a previous report, the coexistence of AAG and Sjögren's syndrome coexistence was found in two patients [38,39]. The frequency of positive anti-gAChR antibody status in large populations or among patients with autonomic neuropathy in Sjögren’s syndrome has not been determined. We believe it is important to clarify the clinical and immunological characteristics of the coexistence of AAG and the other autoimmune diseases. Additionally, attention should be paid to the endocrine disorders that are complications of AAG, because there were five patients (Patients 4, 9, 14, 15, and 19) with endocrine disorders in the study. Three of the five patients who were seropositive for the anti-gAChR antibodies presented with amenorrhea, SIADH, and panhypopituitarism. Several Japanese neurologists have already reported cases of acute pandysautonomia or acute autonomic and sensory neuropathy (AASN) with amenorrhea and/or SIADH [40–42]. They have suggested that patients with autonomic neuropathy might have both peripheral and central nervous system manifestations. The roles of nAChRs are associated with cholinergic neurotransmission, the modulation of dopamine function, the influence of inflammation, and the activity of the hypothalamic-pituitary-adrenal axis [43,44]. We presumed that nAChRs are involved in a variety of neurological systems that are implicated in the pathophysiology of central nervous system involvement including endocrine disorders and psychiatric symptoms.

Ganglionic AChR antibodies have the potential to impair autonomic ganglionic synaptic transmission [9,45,46]. Because both sympathetic and parasympathetic ganglia utilize nicotinic cholinergic synapses, antibodies that interfere with ganglionic transmission could cause pandysautonomia. Our clinical and serological observations suggest that pandysautonomia may be mediated by autoantibodies that interfere with autonomic ganglionic transmission. The patients who were identified in the present study had a failure of both the sympathetic (orthostatic hypotension and anhidrosis) and parasympathetic functions (gut dysmotility, sicca complex, and pupil abnormalities). The autonomic function tests that were performed in this study were incomplete and inadequate, but most of the results (H/M ratio in ^123^I-MIBG myocardial scintigraphy and pupillary response to local instillation) demonstrated postganglionic parasympathetic involvement. Kimpinski et al. characterized the unique sudomotor dysfunction in AAG as widespread, predominantly postganglionic, and a result of lesions at both the ganglia and distal axon [47]. Manganelli et al. have also demonstrated in a sudomotor function study and skin biopsy findings, that there is postganglionic autonomic damage in patients with AAG [48]. They attributed this damage to prolonged and severe impaired synaptic transmission. These reports coincide with our deduction that the anatomic pattern of autonomic dysfunction was predominantly postganglionic.

Another important observation was that the impairment of the autonomic function may be partially reversible in AAG. Patients 1 and 4 (the illustrative cases) improved in response to immunotherapy (plasmapheresis, IVMP, IVIg, and immunosuppressant drugs) with symptomatic therapy. We interpreted this improvement to be related to the correlation with a decrease in the levels of anti-gAChR antibodies in each case. Some patients with seropositive AAG responded to treatment with IVMP, plasmapheresis, or IVIg, and most of these required combined or subsequent treatments to maintain the improvement [49–53]. The more severely affected patients who did not respond to IVMP or PP monotherapy benefited from combined therapy with other first line therapy and immunosuppressant agents, such as prednisolone, azathioprine, and rituximab [54]. They also required prolonged immunotherapy for sustained clinical improvement. These results suggest that antibody production may be ongoing, and not self-limited. Combined treatment with any of the immunosuppressant agents reduced antibody production to levels that were adequate for clinical benefit in our patients. In Patient 4, menstruation restarted after a series of immunotherapy, suggesting an autoimmune mechanism in this process. Thus, immunotherapy can also be efficacious for treatment of the endocrinological abnormalities of patients with AAG.

Some limitations existed in this study. The important limitations of this study were the retrospective design and the small number of subjects. A prospective, multi-center, and clinical observation is necessary to confirm the relationships between the antibody levels, symptoms, and results of the autonomic functions tests. Additional experiments and investigations are necessary to clarify the role of AChR β4 antibodies in the pathogenesis of AAG. In addition, we need to compare the sensitivity and specificity of LIPS with the RIP for the measurement of autoantibodies to gAChR in spiraling numbers.

Our results demonstrated the usefulness of the LIPS assay as a new tool for detecting autoantibodies against gAChR in the patients with AAG. Furthermore, follow-up studies with a greater number of patients with AAG are required to investigate the relationship between the levels of gAChR autoantibodies and clinical features.

## Supporting Information

S1 Table Detailed clinical characteristics of OND patients. The disease control (DC) consisted of 34 subjects with other neurological diseases (OND: mean age, 56.3 ± 20.4 years old, 19 males and 15 females).
(DOCX)

S2 Table Demographic features of patients with subacute AAG/APD and gradual AAG/APD. There was no difference between the demographic features of the seropositive patients in gradual onset and subacute AAG in seropositive patients and no relationship between antibody status and the temporal profile.
(DOCX)
