# Supplementary material for: Clinical Features of Autoimmune Autonomic Ganglionopathy and the Detection of Subunit-Specific Autoantibodies to the Ganglionic Acetylcholine Receptor in Japanese Patients
Source: PLoS One. 2015 Mar 19;10(3):e0118312. doi: 10.1371/journal.pone.0118312 (PMC4366081; doi:10.1371/journal.pone.0118312)
Supplement: S1 Table — The disease control (DC) consisted of 34 subjects with other neurological diseases (OND: mean age, 56.3 ± 20.4 years old, 19 males and 15 females). (DOCX) [file pone.0118312.s001.docx]

S1 Table. Detailed clinical characteristics of OND patients

|  | Subacute patients with AAG/APD  Anti-gAChR Ab positive |
| --- | --- |
| Amyotrophic lateral sclerosis | 1 |
| Amyloidosis | 5 |
| Diabetic neuropathy | 5 |
| Hereditary motor sensory neuropathy | 2 |
| Horner syndrome | 1 |
| Hypokalemic myopathy | 1 |
| Movement diorders | 3 |
| Multiple system atrophy | 6 |
| Multiple cranial neuropathy | 1 |
| Myotonic dystrophy | 1 |
| Narcolepsy | 1 |
| Neuroblastoma | 1 |
| Parkinson’s disease | 5 |
| Temporal epilepsy | 1 |

The disease control (DC) consisted of 34 subjects with other neurological diseases (OND: mean age, 56.3 ± 20.4 years old, 19 males and 15 females).
