# Supplementary material for: Clinical Features of Autoimmune Autonomic Ganglionopathy and the Detection of Subunit-Specific Autoantibodies to the Ganglionic Acetylcholine Receptor in Japanese Patients
Source: PLoS One. 2015 Mar 19;10(3):e0118312. doi: 10.1371/journal.pone.0118312 (PMC4366081; doi:10.1371/journal.pone.0118312)
Supplement: S2 Table — There was no difference between the demographic features of the seropositive patients in gradual onset and subacute AAG in seropositive patients and no relationship between antibody status and the temporal profile. (DOCX) [file pone.0118312.s002.docx]

S2 Table. Demographic features of patients with subacute AAG/APD and gradual AAG/APD

|  | Subacute patients with AAG/APD  Anti-gAChR Ab positive | Gradual patients with AAG/APD  Anti-gAChR Ab positive | P value |
| --- | --- | --- | --- |
| Number of patients | 9 | 15 |  |
| Age (yr) | 42.1 ± 22.3 | 57.8 ± 17.3 | 0.066 |
| Age at onset (yr) | 42.1 ± 22.3 | 49.5 ± 20.1 | 0.417 |
| Sex (female, %) | 4 (44.4) | 9 (60.0) |  |
| Antecedent event (%) | 3 (33.3) | 1 (6.7) | 0.106 |
| Orthostatic hypotension and/or orthostatic intolerance (%) | 9 (100.0) | 11 (73.3) | 0.062 |
| Sicca complex (%) | 6 (66.7) | 8 (53.3) | 0.553 |
| Coughing episodes (%) | 1 (11.7) | 3 (20.0) | 0.612 |
| Heat intolerance and/or anhidrosis (%) | 7 (77.8) | 8 (53.3) | 0.256 |
| Pupil abnormality (%) | 6 (66.7) | 5 (33.3) | 0.129 |
| Gastrointestinal tract symptoms (%) | 8 (88.9) | 14 (93.3) | 0.756 |
| Bladder dysfunction (%) | 5 (55.6) | 11 (73.3) | 0.401 |
| Sexual dysfunction^a^ (%) | 2 (40.0) | 5 (71.4) | 0.432 |
| Anti-gAChRα3 Ab (A.I., LIPS) | 2.057 ± 1.906 | 2.071 ± 0.968 | 0.404 |
| Anti-gAChRβ4 Ab (A.I., LIPS) | 0.653 ± 0.319 | 0.910 ± 0.473 | 0.165 |

1. We reviewed the 26 male patients only.

There was no difference between the demographic features of the seropositive patients in gradual onset and subacute AAG in seropositive patients and no relationship between antibody status and the temporal profile.
